# Supplementary material for: A Critical View of the Application of the APEX Software (Aqueous Photochemistry of Environmentally-Occurring Xenobiotics) to Predict Photoreaction Kinetics in Surface Freshwaters
Source: Molecules. 2019 Dec 18;25(1):9. doi: 10.3390/molecules25010009 (PMC7017383; doi:10.3390/molecules25010009)
Supplement: Supplementary file 1 [file molecules-25-00009-s001.zip › 3.2.4_gcc-4.4.0.zip!/3.2.4_gcc-4.4.0/3.2.4_gcc-4.4.0/doc/octave/html/liboctave/Objective-Functions.html]

Objective Functions - Octave C++ Classes


Next: Bounds,
Previous: Optimization,
Up: Optimization


---

### 9.1 Objective Functions

— : **Objective** (void)  
— : **Objective** (const objective\_fcn)  
— : **Objective** (const objective\_fcn, const gradient\_fcn)  
— : **Objective** (const Objective &a)

— : Objective& **operator =** (const Objective &a)

— : objective\_fcn **objective\_function** (void) const;

— : Objective& **set\_objective\_function** (const objective\_fcn)

— : gradient\_fcn **gradient\_function** (void) const;

— : Objective& **set\_gradient\_function** (const gradient\_fcn)
